# Microbial induced calcium carbonate precipitation under extreme environmental conditions: impacts on biomineralization process and mechanical performance of concrete

**DOI:** 10.3389/fbioe.2026.1808242

**Published:** 2026-06-10

**Authors:** Mai M. Abdelwahed, Amany M. Reyad, Ahmed. Abd-Alazim, Gehad Mokhtar

**Affiliations:** 1 Department of Civil Engineering, Faculty of Engineering, Fayoum University, Fayoum, Egypt; 2 Department of Botany, Faculty of Science, Fayoum University, Fayoum, Egypt; 3 Future high Institute of Engineering in Fayoum, Fayoum, Egypt

**Keywords:** *Bacillus* paramycoides, biomineralization, compressive strength, MICP, nano eggshells, pH, salinity

## Abstract

**Introduction:**

Cracks in concrete structures can significantly decrease their lifespan by exposing reinforcement to the outside environment, leading to concrete degradation. To address this issue, bio-healing techniques have been developed, including biomineralization, where bacteria are employed to initiate microbially induced calcium precipitation (MICP).

**Methods:**

This study examines the mechanisms of bio-healing in concrete under extreme environmental conditions, focusing on variations in pH and salinity. It utilizes immobilized cells of *Bacillus paramycoides strain G10* for this investigation. Mineralization and the concrete strength were investigated using five distinct pH values (3.0, 5.0, 7.0, 9.0, and 11.0) and various salinity levels (0, 1, 2.5, and 5%).

**Results:**

At very high pH, rapid urea hydrolysis leads to the precipitation of mixed phases such as calcite and amorphous calcium carbonate, while soluble ions also promote the growth of long ettringite needles that help fill cracks. On the other hand, precipitation usually shifts toward metastable polymorphs such as aragonite or vaterite, or even amorphous phases with distorted and irregular morphologies, under extremely high salinity, which inhibits the production of stable calcite. Compressive strength improvements in 15 specimens containing bacteria immobilized on nano eggshells at pH 3, 5, 7, 9, and 11 were 25.5, 15.2, 13.5, 16.2, and 17.6%, respectively, in comparison with the corresponding control specimens without bacteria cured under identical conditions. Under various salinity concentrations of 0, 1, 2.5, and 5%, improvements were 5.2, 1.4, 3.3, and 6.7%, respectively, for specimens containing bacteria immobilized on nano eggshells when compared to their relative controls.

**Discussion:**

The combined action of calcite, which provides long-term stability, and ettringite, which offers early crack filling, enhances the strength and durability of concrete, making pH control essential in MICP systems. Understanding the interplay between environmental factors and bacterial sustainability is crucial in enhancing concrete infrastructure.

## 1 Introduction

In the current era of continuous urbanization, concrete stands out as an exceptionally versatile and valuable product (Palanisamy et al., 2020). It is susceptible to issues such as settlement, cracking, premature drying, and other conditions (Jena et al., 2020a; Yoo et al., 2019). These cracks may form a continuous network, which results in increased concrete permeability. The severity of cracks can differ from one structure to another (Roig-Flores et al., 2015). As a result, numerous research projects are moving forward to increase the endurance of civil constructions and identify environmentally acceptable, highly effective repair materials. The use of bacterial concrete to repair cracks has drawn a lot of interest recently (Jonkers et al., 2010; Van Tittelboom et al., 2010; Walraven and Stoelhorst, 2000). The importance of bacteria-induced calcium carbonate precipitation has driven the increasing application of biomineralization techniques in recent years (De Muynck et al., 2010a; Soon et al., 2014). According to the studies, bio-deposited minerals like calcium carbonate are more environmentally friendly and more acceptable as building materials (Jonkers et al., 2010). In cement-based materials, varying environmental conditions frequently affect the bacterial-based bio-healing effect. The ability of bacteria to survive in unfavorable environmental conditions is crucial after determining which metabolic pathway will mediate the process in terms of effective extracellular production of carbonate ions as well as investigating all possible effects of bacteria during the bio-healing effect (Soon et al., 2014). The main challenge in applying bio-healing concrete is achieving uniform treatment and reliable monitoring under real conditions. Bacterial activity, essential for crack repair, may decline over time due to environmental factors (pH, temperature, salinity, porosity, nutrients, etc.) (Anbu et al., 2016).

Concrete initially provides a highly alkaline environment (pH 12.5–13); however, this alkalinity diminishes over time as a result of deterioration mechanisms such as acid attack, carbonation, and chloride ingress, potentially reducing the pH to below 9 (Arunachalam et al., 2010). The pH level and its variations will affect metabolic activity, particularly enzymatic activity. The bacterial-induced calcium precipitation process can occur in alkaline environments because the high pH value promotes the formation of carbonate and bicarbonate ions. The ideal pH value for metabolic activity and the bio-healing effect is frequently the same since the alkaline byproducts are produced during carbonate saturation (Soon et al., 2014). The successful development of stable crystalline forms like calcite and aragonite is best facilitated by a pH value over 9.5 (Anbu et al., 2016), but a pH drop may result from CaCO_3_ precipitation (Arunachalam et al., 2010). At the onset of the bio-healing action, Gat et al. (2014) demonstrated that bacteria may change the pH value by nearly two pH units during CaCO_3_ precipitation, with quick and sudden shifts towards a very alkaline matrix. Accordingly, the bio-healing effect would favor bacteria that are alkalotolerant or alkalophilic. Bacterial-induced calcium precipitation has an acceptable potential in the bio-healing effect, and alkalophilic bacteria develop best at pH levels around 10 (Rampelotto, 2010; Šovljanski et al., 2021).

Cracks in marine environments appear in concrete due to penetration of chloride and sulfate ions. The chloride concentration in the cracks is similar to that on the outer concrete surface exposed to the chloride environment. The repercussion of this process can be the promoted corrosion of rebar and the destructive process of the infrastructure unit (Khan et al., 2021). One of the main environmental elements affecting bacterial activity in bio-healing concrete is salinity. Bacterial cells undergo osmotic stress in saline environments, such as coastal locations, subterranean buildings where groundwater intrusion occurs, and marine structures, which can lower their urease efficiency and metabolic activity. Excessive sodium chloride (NaCl) concentrations can change the rate and shape of CaCO_3_ crystal formation, restrict nutrient intake, and decrease bacterial growth. As a result, in saline environments, bio-concrete’s healing effectiveness may be significantly decreased. Therefore, to improve concrete healing, understanding the relationship between salt and bacterial bio-healing mechanisms is essential (Javeed et al., 2024).

The main objective of this study is to utilize bacteria immobilized on eggshell nanoparticles as a healing agent in concrete in order to investigate the influence of external environmental factors such as salt concentration (NaCl) and pH variations on the efficiency of bacterial bio-healing. Studying the impact of salinity and pH on bacterial viability, crystal morphology, and crack-sealing efficiency will provide valuable insights for optimizing mix design and ensuring long-term durability of bio-concrete in aggressive environments such as marine and coastal structures.

Nano-eggshell particles have recently emerged as promising sustainable materials in cement-based and bio-concrete systems due to their high calcium carbonate (CaCO_3_) content, low cost, and eco-friendly nature. Their nanoscale size enhances the packing density of the cement matrix, improves hydration reactions, and reduces porosity, leading to significant improvements in mechanical properties and durability. In addition, nano-eggshell particles act as effective nucleation sites that facilitate microbially induced calcium carbonate precipitation (MICP), thereby accelerating crack healing and enhancing interfacial bonding within the cementitious matrix. Recent studies have confirmed that eggshell-derived nanoparticles can significantly improve compressive, tensile, and flexural strength when used as bacterial carriers or supplementary cementitious materials, due to their dual role as calcium sources and micro-fillers (Abdelwahed et al., 2025). Therefore, nano-eggshell particles represent a sustainable and efficient approach for improving the performance and self-healing capacity of bacterial concrete systems.

In our study, nano eggshell units are selected as a natural and sustainable carrier material due to their high calcium content and porous structure, which can protect bacterial cells and enhance their survival inside the concrete matrix. Nanomaterials exhibit new properties, including microscopic size effects, surface effects, filling the internal pores and making the paste denser; and adsorption properties that macroscopic materials lack (Ansari et al., 2016). They may control the hydration process of cement, improving the mechanical qualities and endurance of the hardened paste (Sukanya and Prabhakar, 2017). These compounds may successfully preserve microorganisms in the cement matrix due to their surface interactions and increased surface area (Bhavsar, 2023). concrete bio-healing, most have focused on neutral or mildly stressful conditions, single environmental factors, or nonimmobilized bacterial systems. A systematic examination of the combined effects of extreme pH and salinity on MICP-particularly using bacteria immobilized on nano-sized particles remains scarce. Moreover, few studies have concurrently analyzed the resulting crystal morphologies (calcite, aragonite, vaterite) and secondary mineral phases, such as ettringite. In the present study, we aim to fill this research gap by evaluating how *Bacillus paramycoides* strain G10, when immobilized, mediates mineralization and enhances concrete strength under varying environmental conditions. This integrated investigation provides novel insights into bio-healing mechanisms by linking environmental stressors, bacterial viability, mineral formation, and mechanical performance of concrete.

## 2 Materials and methods

### 2.1 Microbially induced CaCO_3_ precipitation at various pH values

A technique based on the procedure proposed by Krishnapriya et al. (2015) was adopted to quantify calcium carbonate precipitation. A bacterial suspension (2%) was added to 30 mL of NB-U/Ca medium, which consisted of nutrient broth supplemented with 2% urea and CaCl_2_. The samples were incubated on a shaker at 130 rpm and 30 °C for 7 days. The experiment was conducted under different pH conditions ranging from 3 to 11 and salinity levels of 0, 1, 2.5, and 5%. At the end of the incubation period, the formed CaCO_3_ precipitate was collected by filtration using Whatman filter paper, dried in an oven at 55 °C for 8 h, and weighed. The amount of calcium carbonate (Wc) was determined using the following equation:

Wc=Wfc−Wf

where Wfc represents the weight of the filter paper with precipitate, and Wf is the weight of the empty filter paper.

### 2.2 Materials

Ordinary Portland cement (OPC), supplied by Wadi El-Nile Cement Company in Beni Suef, Egypt, was the type of cement utilized in this investigation. The mortar specimens were prepared using natural sand from quarries located on October 6. Natural aggregate (basalt) became the coarse aggregate in this study. *Bacillus paramycoides* strain G10 (GenBank accession number: MZ430955) (Krishnapriya et al., 2015) was the bacterium employed in this investigation. Eggshell nanoparticles (EShN), which were chosen for their capacity to precipitate calcium carbonate through microbially induced calcite precipitation (MICP), were made and employed as carrier units for bacterial immobilization; freshwater was utilized for mixing and curing, except in cases when salinity adjustments were necessary. Saline curing solutions were made with sodium chloride (analytical grade, NaCl). Appropriate acids or bases were added to the water to change its pH.

### 2.3 Preparation of eggshells nanoparticles (EShN)

Eggshell trash was supplied from chicken farms in El Azab, Fayoum, Egypt. Once the eggshell waste was cleansed with tap water, it was exposed to the sun for a few days to dry. Before being run through 100 μm sieves, the eggshells were broken and ground into a powder. For 24 h, eggshell units were dried in an oven set between 100–110 °C to remove any last traces of moisture. Following drying, eggshell nanoparticles (EShN) were produced by grinding them in a ball mill for about 90 min and then in a disc mill for another 10 min (Muñoz et al., 2007; Odo et al., 2012).

### 2.4 Preparation of bacterial cells and immobilization

**FIGURE 1:**
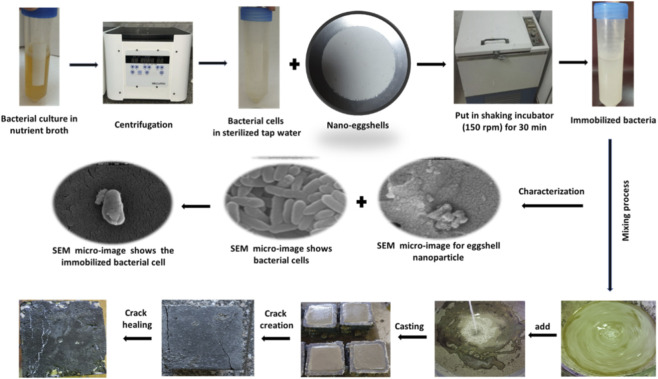
Schematic diagram of the experimental procedure for preparing immobilized bacterial cells and evaluating the bio-healing performance in concrete. Bacterial cells were cultured, immobilized onto nano-eggshell carriers, and characterized via SEM imaging. The immobilized formulation was added into concrete during casting, followed by controlled crack induction and subsequent evaluation of healing performance.

In this study, a bacterium, *Bacillus paramycoides* strain G10 (GenBank accession number: MZ430955), was used (Mokhtar et al., 2021). The bacterial strain was cultured for 48 h in a nutrient broth to produce cell density. To collect the bacterial cells, several centrifugation procedures were carried out with dual-sterilized tap water. By employing the force of electrostatic attraction, EShN was utilized to immobilize the bacterial cells. EShN (125 g/L) was dispersed in distilled water using a sonicator set to 500 W for 2 minutes. After that, the bacterial cells were added and shaken in an incubator at 30 °C and 150 rpm for 30 min, as shown in (Figure 1).

### 2.5 Experimental design

The concrete mixture design is presented in Table 1. The mixtures were designed, and a testing program was conducted in accordance with ECP (Egyptian Code of Practice ECP 203-18, 2018). Eggshell nanoparticles were used as bacterial carriers to immobilize 1.3 × 10^7^ cells/cm^3^ of bacterial cells in mixtures. The nutrients 2 g of yeast extract, 65 g of urea, and 40 g of anhydrous calcium chloride were dissolved in sterilized distilled water for mortar and concrete mixtures to use to produce the bio-based concrete. The mixture was then shaken for 2 minutes, and a bacterial cell solution and 17.5 kg/m^3^ of eggshell nanoparticles were added. Standard cubic specimens (150 × 150 mm) were cast for compressive strength testing. For flexural strengths, the indirect splitting tensile strength beam (100 × 100 × 500 mm) and cylinder (150 × 300 mm) were tested for hardened concrete. The specimens were demolded and cured in various environmental settings following a 24 h casting period. The compressive, flexural, and indirect tensile strength tests were carried out for hardened concrete at 28 days. The results indicated the formation of cracks under the applied conditions.

**TABLE 1:** Concrete mixtures design.

Mixture	Water (kg/m^3^)	Cement (kg/m^3^)	Sand (kg/m^3^)	Coarse aggregate (Kg/m^3^)	Egg shells nanoparticles weight (kg/m^3^)	Bacterial cells	Type of mixtures
C	140	332.5	675.5	1340.2	17.5	--	Concrete
CB	140	332.5	675.5	1340.2	17.5	bacterial cells	Bio-concrete

C: refer to the concrete mixture without bacteria.

CB: refer to the concrete mixture with bacterial cells immobilized on nanoparticles.

### 2.6 Environmental exposure conditions

#### 2.6.1 pH exposure

To evaluate the influence of pH, curing solutions were prepared with five distinct pH values: 3.0, 5.0, 7.0, 9.0, and 11.0. The adjustment of pH was achieved using 1 M HCl (for acidic conditions) and 1 M NaOH (for alkaline conditions). Specimens were immersed in these solutions for 90 days.

#### 2.6.2 Salinity exposure

To assess the effect of salinity, specimens containing immobilized bacterial cells were cured in NaCl solutions with concentrations of 0%, 1%, 2.5% and 5% by weight. The control curing condition consisted of fresh water. The curing duration was 90 days.

### 2.7 Statistical analysis

Statistical analysis was performed using GraphPad Prism software. All experiments were conducted in triplicate, and the results are expressed as mean ± standard deviation (SD). Statistical comparisons between groups were carried out using one-way analysis of variance (ANOVA) followed by an appropriate *post hoc* test to determine significant differences among groups. A p-value of less than 0.05 was considered statistically significant. In the graphical representations, error bars indicate the standard error (SE) of the mean values. Different letters (e.g., a, b, c) above the columns denote statistically significant differences between groups, where columns sharing the same letter are not significantly different, while those with different letters indicate significant differences at p < 0.05.

## 3 Results

### 3.1 Microbially induced CaCO_3_ precipitation at various pH values

**FIGURE 2:**
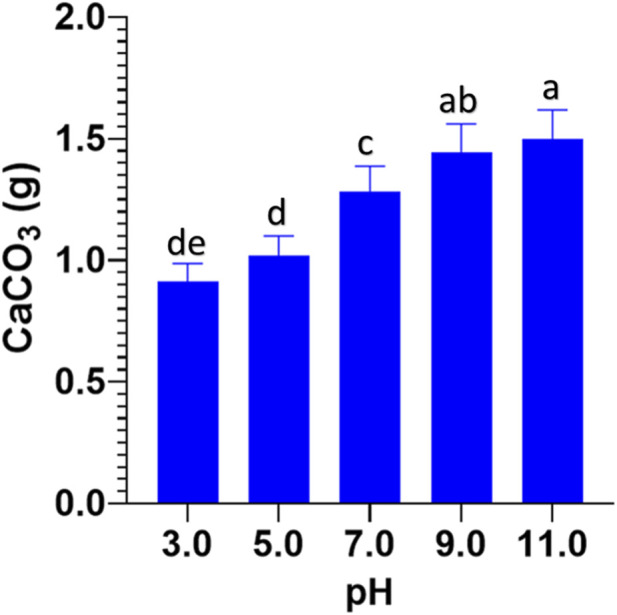
Influence of pH levels on calcium carbonate precipitation.

The relationship between pH levels and the amount of calcium carbonate (CaCO_3_) formed indicates that, as the pH increases from 3 to 11, a gradual rise in CaCO_3_ precipitation is observed (Figure 2). Lower precipitation values are recorded under acidic conditions, while higher amounts are obtained in neutral and alkaline environments. The highest CaCO_3_ yield is achieved at pH 11, indicating that alkaline conditions promote calcium carbonate formation. It was observed that CaCO_3_ precipitation increased by 12.5% and 16.4% under curing conditions at pH 9 and pH 11, respectively.

**FIGURE 3:**
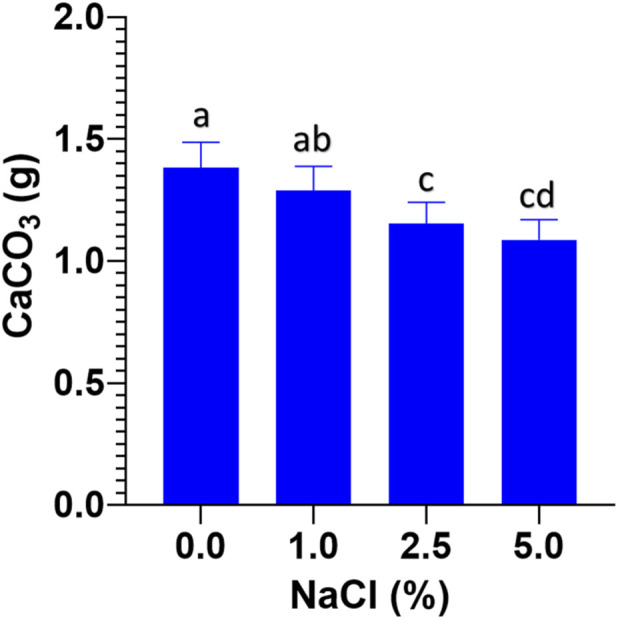
Influence of NaCl concentration on calcium carbonate precipitation.

The influence of salinity on calcium carbonate (CaCO_3_) precipitation at different NaCl concentrations (0, 1, 2.5, and 5%) indicates a decreasing trend in CaCO_3_ formation with increasing salt content (Figure 3). The highest amount of CaCO_3_ is observed in the absence of NaCl (0%), while the lowest precipitation occurs at 5% NaCl. Overall, the figure demonstrates that elevated salinity levels negatively affect calcium carbonate precipitation. It was observed that CaCO_3_ precipitation decreased by 6.5, 16.6, and 22% under curing conditions at NaCl concentrations 1, 2.5, and 5%, respectively.

### 3.2 Environmental exposure conditions

#### 3.2.1 pH exposure

**FIGURE 4:**
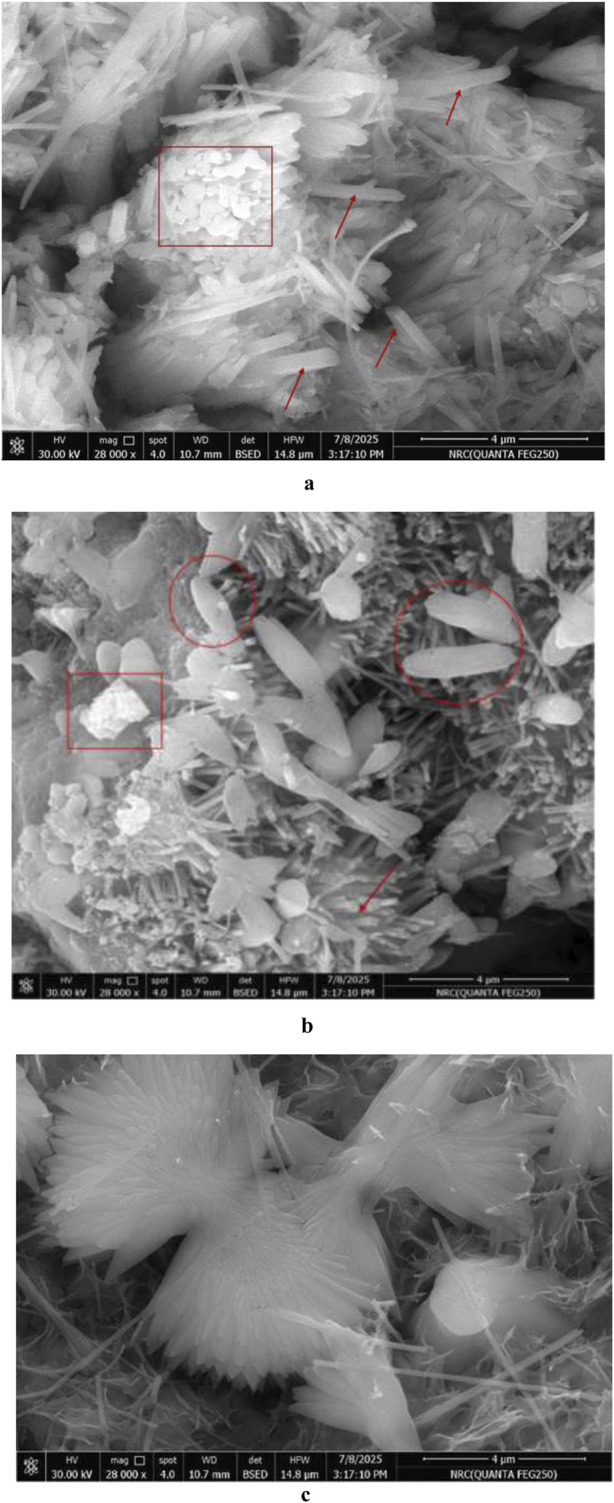
SEM micro-images for various pH levels. **(a)** neutral conditions, **(b)** alkaline conditions, and **(c)** acidic conditions. **(a)** High-resolution SEM micrograph at neutral conditions illustrating the co-precipitation of mineral phases during MICP. Dense, acicular ettringite needles form an interwoven matrix (red arrows). The red square highlights embedded calcium carbonate aggregates indicating concurrent CaCO3 nucleation within the ettringite framework and confirming synergistic mineralization under the tested conditions. SEM image acquired at 30 kV and 28,000× magnification (WD = 10.7 mm, spot size = 4.0) using a BSED detector; HFW = 14.8 µm; scale bar = 4 μm. **(b)** SEM micrograph at high pH showing the morphology of the synthesized material. Rod bacterial cells are clearly visible (circled). well-formed rhombohedral calcite crystals (squared), and the red arrow indicates a more developed ettringite network.b. SEM micrograph at high pH showing the morphology of the synthesized material. Rod bacterial cells are clearly visible (circled). well-formed rhombohedral calcite crystals (squared), and the red arrow indicates a more developed ettringite network. SEM image acquired at 30 kV and 28,000× magnification (WD = 10.7 mm, spot size = 4.0) using a BSED detector; HFW = 14.8 µm; scale bar = 4 μm. **(c)** SEM micrograph showing well-defined flower-like crystalline clusters from calcite Fine and thin ettringite was detected. SEM image acquired at 30 kV and 28,000× magnification (WD = 10.7 mm, spot size = 4.0) using a BSED detector; HFW = 14.8 µm; scale bar = 4 µm.

Moderately developed calcium carbonate (CaCO_3_) crystals with irregular morphologies and small sizes are present in the bacterial concrete microstructure at neutral pH 7 (Figure 4). Under neutral conditions, ettringite crystals appear as moderately thick needles with a uniform distribution, indicating balanced mineral development. At high pH, the microstructure gets more compacted and denser. Long, thick, needle-like ettringite that links to form a continuous network is apparent; also, well-formed rhombohedral calcite crystals can be seen. At low pH, significant changes in surface morphology occur. The calcium carbonate crystals exhibit disordered, cauliflower-like shapes. Ettringite formation is limited, appearing as short, thin, and incomplete needles.

#### 3.2.2 Salinity exposure

**FIGURE 5:**
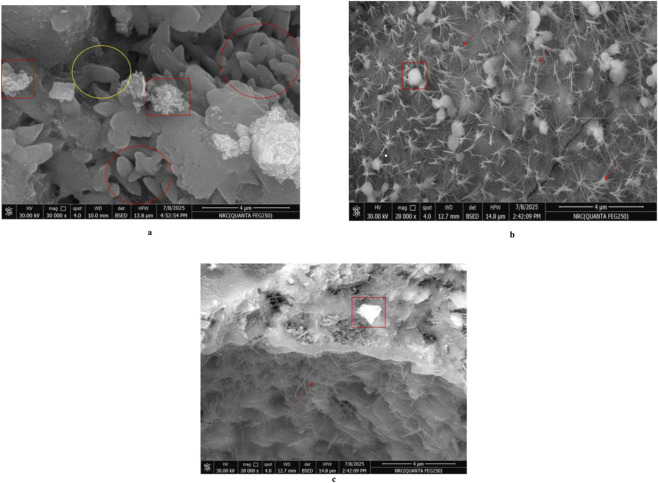
SEM micro-images for salinity levels. **(a)** at 1% NaCl, **(b)** at 2.5% NaCl, and **(c)** at 5% NaCl. **(a)** High-magnification SEM micrograph under 1% NaCl illustrating the mineralization products formed during MICP. Distinct calcium carbonate morphologies are observed (the red squares), Cell-shaped bacteria (red circles), and the immobilized bacterial cells were detected in concrete matrix (yellow circle). SEM image acquired at 30 kV and 30,000× magnification (WD = 10.0 mm, spot size = 4.0) using a BSED detector; HFW = 13.8 µm; scale bar = 4 μm. **(b)** SEM analysis for 2.5% NaCl. ettringite structures are radially oriented acicular (indicated by arrows). Spherical cores (squared) represent calcite. SEM image acquired at 30 kV and 28,000× magnification (WD = 12.7 mm, spot size = 4.0) using a BSED detector; HFW = 14.8 µm; scale bar = 4 μm. **(c)** SEM micro-image shows the calcite and ettringite structures at 5% NaCl. Square indicates for calcite and arrow represents ettringite. SEM image acquired at 30 kV and 28,000× magnification (WD = 12.7 mm, spot size = 4.0) using a BSED detector; HFW = 14.8 µm; scale bar = 4 µm.

Calcite is primarily observed as rhombohedral crystals at 1% NaCl (Figure 5). The overall structure is found to be stable, relatively dense and compact, with a clear bacterial biomineralization appearance, where bacterial cells persist and are observed within the concrete matrix. At 2.5% NaCl, calcite typically forms aggregated clusters rather than distinct rhombohedral crystals. Ettringite crystals display lower aspect ratios and become short branches like structures. At 5% NaCl, more irregularities are formed in the crystallization process. Ettringite develops as distorted and loosely packed needles. Calcite typically precipitates slowly in regular aggregates. The resulting microstructure is compact but highly heterogeneous, reflecting rapid precipitation under elevated ionic stress.

### 3.3 Compressive strength

#### 3.3.1 pH

**FIGURE 6:**
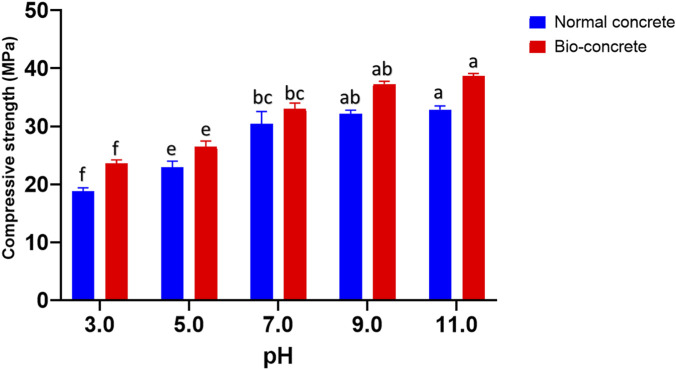
Shows the comparison of compressive strength of normal concrete and bio-concrete at different pH levels (3–11). Data are presented as mean ± standard error (n = 3).

The experimental results indicated that specimens incorporating bacteria immobilized on nano eggshells exhibited an increase in compressive strength when cured in water at pH levels of 3, 5, 7, 9, and 11, compared to the corresponding control specimens without bacteria under the same conditions (relative controls). Compressive strength improvement of specimens containing bacteria immobilized on nano eggshells is observed at pH 3, 5, 7, 9, and 11 by 25.5%, 15.2%, 13.5%, 16.2%, and 17.6%, respectively, compared with the corresponding control specimens without bacteria cured under identical conditions (relative controls) (Figure 6). The results showed that the compressive strength for specimens incorporating bacteria immobilized on nano eggshells was reduced by 28.8% and 20.3% when cured at pH 3 and pH 5, respectively. Conversely, an enhancement of 12.5% and 16.7% was observed at pH 9 and pH 11, respectively, in comparison with the reference specimens cured at neutral pH 7.

#### 3.3.2 Salinity

**FIGURE 7:**
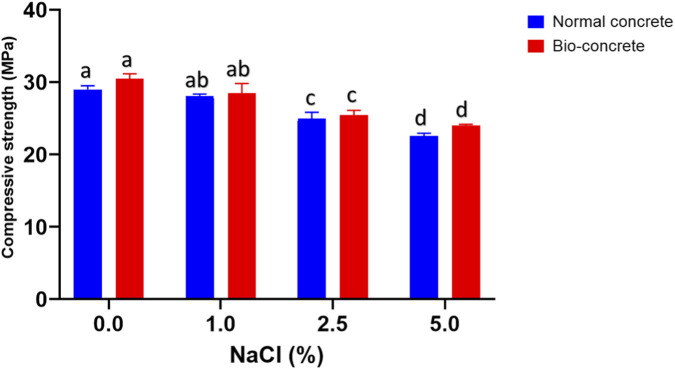
Shows the comparison of compressive strength of normal concrete and bio-concrete at different NaCl concentration (0–5)%. Data are presented as mean ± standard error (n = 3).

The results of the study showed that specimens with immobilized bacterial cells cured at salinity concentrations of 0, 1, 2.5, and 5% had a higher compressive strength than their corresponding control specimens without bacteria subjected to the same salinity concentrations. The compressive strength increased by 5.2%, 1.4%, 3.3%, and 6.7% for specimens containing bacteria immobilized on nano eggshells and cured at salinity concentrations of 0, 1, 2.5, and 5%, respectively, compared with their corresponding control specimens without bacteria subjected to the same salinity conditions (Figure 7). The compressive strength decreases for the concrete specimens supplemented with immobilized bacterial cells when they were cured at salt concentrations of 1, 2.5, and 5% by 6.6, 16.7, and 31.3%, respectively, as they were compared to the control sample (without NaCl).

### 3.4 Flexure and tensile strength

#### 3.4.1 pH

**FIGURE 8:**
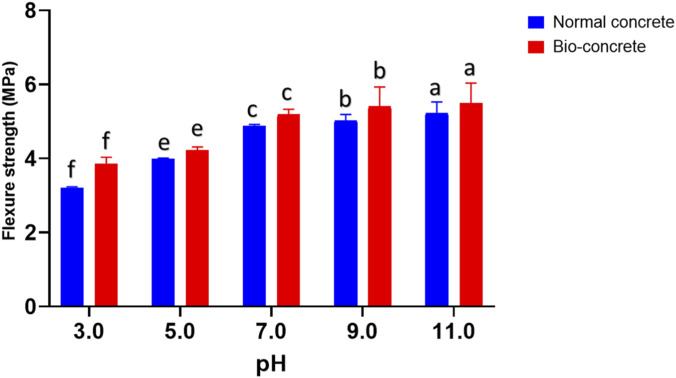
Shows the comparison of flexure strength of normal concrete and bio-concrete at different pH levels (3–11). Data are presented as mean ± standard error (n = 3).

**FIGURE 9:**
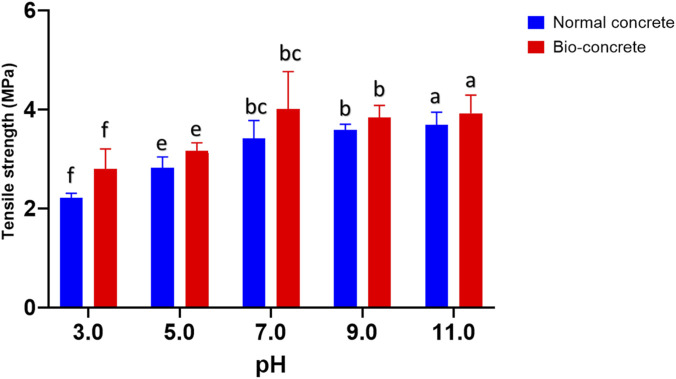
Shows the comparison of tensile strength of normal concrete and bio-concrete at different pH levels (3–11). Data are presented as mean ± standard error (n = 3).

It was observed that the flexure and tensile strength increased for the concrete containing immobilized bacterial cells when they were cured at pH 3, 5, 7, 9, and 11 compared to their relative controls, which were without bacteria and subjected to the same conditions. For the specimens containing bacteria immobilized on nano eggshells, the flexure and tensile strength improved when they were cured at pH 9 and 11 and decreased when they were cured at pH 3 and 5 in comparison to those that were cured at pH 7. The flexure strength improvements of specimens containing bacteria immobilized on nano eggshells at pH 3, 5, 7, 9 and 11 were 19.9, 6, 6.4, 5.7, and 5%, respectively, in comparison with the corresponding control specimens without bacteria cured under identical conditions (relative controls). For the specimens containing bacteria immobilized on nano eggshells, the flexure strength improved when they were cured at pH 9 and 11 by 4.2% and 5.8%, respectively, and decreased when they were cured at pH 3 and 5 by 25.8% and 18.7%, respectively, in comparison to those that were cured at pH 7 (Figure 8). Tensile strength improvements of specimens containing bacteria immobilized on nano eggshells are observed at pH 3, 5, 7, 9, and 11 by 26.7%, 15.6%, 7.9%, 7.3%, and 6.2%, respectively, compared with the corresponding control specimens without bacteria cured under identical conditions (relative controls) (Figure 9). The data demonstrates that tensile strength decreased by 23.9% and 11.4% at pH 3 and 5, respectively. while it increased by 4.3% and 6.5% at pH 9 and 11 for the specimens with bacteria immobilized on nano eggshells, compared to the neutral condition at pH 7.

#### 3.4.2 Salinity

**FIGURE 10:**
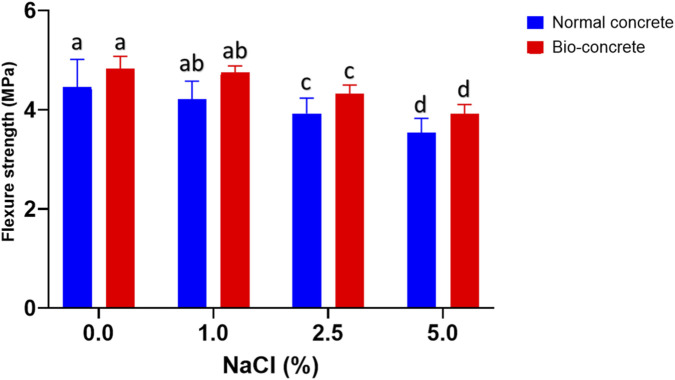
Shows the comparison of flexure strength of normal concrete and bio-concrete at different NaCl concentration (0–5)%. Data are presented as mean ± standard error (n = 3).

It was noted that the flexure strength and tensile strength increased for the specimens containing immobilized bacterial cells when they were cured at salinity concentrations of 1, 2.5, and 5% compared to the corresponding control specimens without bacteria cured under identical conditions (relative controls). Flexure strength increased for concrete specimens supplemented with immobilized bacterial cells when cured at salinity concentrations of 0, 1, 2.5, and 5% by 8.1%, 12.8%, 10.2%, and 10.5%, respectively, compared with their corresponding control specimens without bacteria subjected to the same salinity conditions (Figure 10). As illustrated in the figure, concrete specimens containing immobilized bacterial cells exhibited reductions in flexural strength of 1.5, 10.4, and 18.9% when exposed to salt concentrations of 1, 2.5, and 5%, respectively, relative to the control specimen cured without salinity.

**FIGURE 11:**
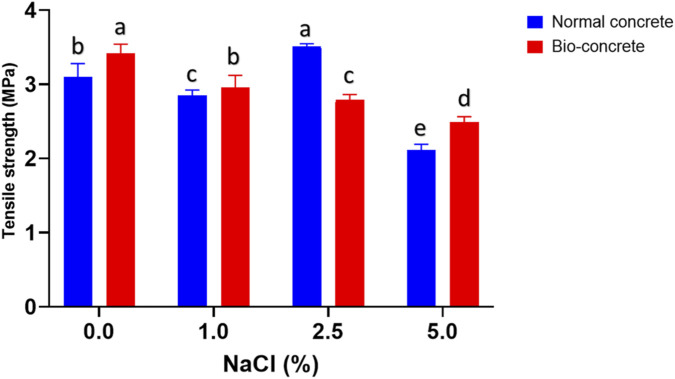
Shows the comparison of tensile strength of normal concrete and bio-concrete at different NaCl concentration (0–5)%. Data are presented as mean ± standard error (n = 3).

The tensile strength of concrete specimens containing immobilized bacterial cells improved by 10.3%, 3.9%, 10.8%, and 18% at salinity concentrations of 0, 1, 2.5, and 5%, respectively, compared with their corresponding control specimens without bacteria cured under identical conditions (Figure 11). In contrast, the control specimens without bacteria exhibited reductions in tensile strength of 13.5, 18.7, and 27.2% at salinity concentrations of 1, 2.5, and 5%, respectively, relative to the specimen cured without salinity.

### 3.5 EDX elemental analysis for calcite crystals, ettringite, and bacterial cell structure

**FIGURE 12:**
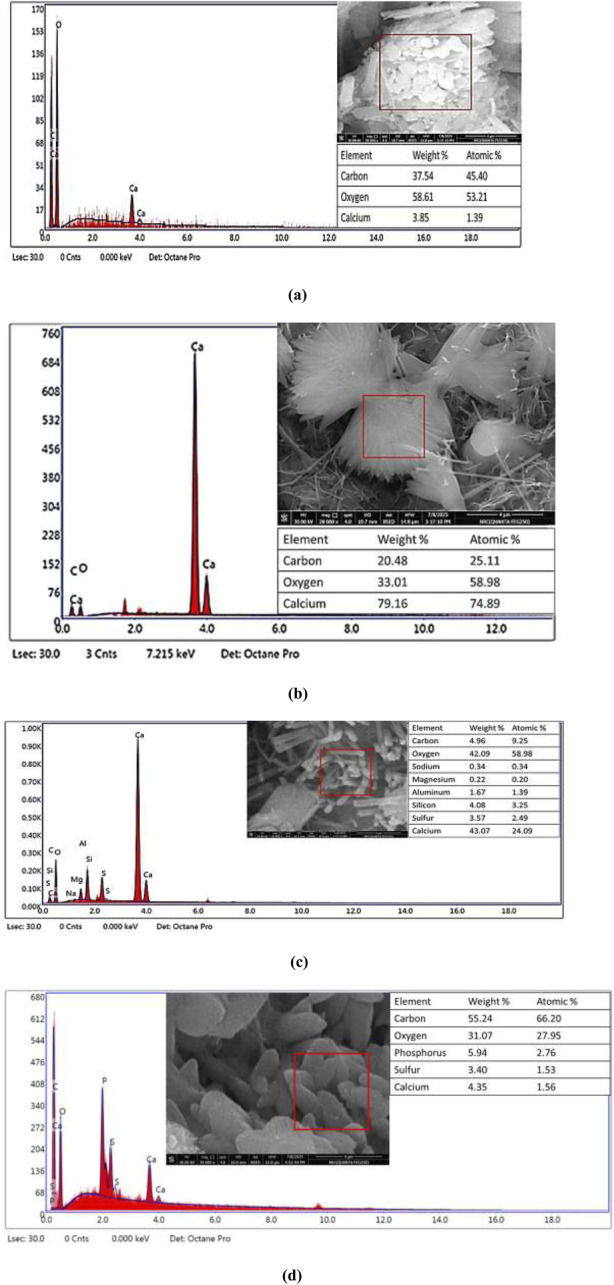
SEM–EDX analysis shows elemental spectra of calcite crystals **(a,b)** ettringite structures **(c)** and bacterial cell structure **(d)**.

As shown in Figure 12, energy-dispersive X-ray spectroscopy (EDX) analysis was performed to determine the elemental composition of the formed calcite crystals, ettringite structures, and bacterial cell morphology. The EDX spectra and corresponding elemental distribution confirmed the presence of major elements including carbon (C), oxygen (O), calcium (Ca), phosphorus (P), and sulfur (S), indicating the formation of calcite and ettringite phases associated with bacterial activity.

The elemental composition of calcite crystals showed a high percentage of carbon and oxygen with a significant calcium signal, confirming calcium carbonate precipitation. Additionally, sulfur and phosphorus peaks were detected in the ettringite structures, supporting the formation of sulfate-based mineral phases. The bacterial cell structure exhibited elemental signals mainly composed of carbon and oxygen with minor calcium and phosphorus, reflecting the biological matrix and biomineralization process.

## 4 Discussion

### 4.1 Environmental exposure conditions

#### 4.1.1 pH exposure

At pH 7, the solution is neutral; therefore, CaCO_3_ precipitation proceeds relatively slowly. This reduces the concentration of carbonate ions and lowers supersaturation (Seifan et al., 2017). Calcite, which is the most stable polymorph of calcium carbonate, forms with irregular morphologies and variable crystal sizes. These conditions increase chances of metastable phases like aragonite and vaterite (Hanifa et al., 2014; Rodriguez-Blanco et al., 2012; Said et al., 2025). The slower nucleation rate at pH 7 promotes imperfect and discontinuous crystal growth, leading to porous and loosely packed structures rather than dense, well-faceted calcite. Because the precipitated CaCO_3_ crystals are less compact and less effective at sealing voids, this phenomenon in bacteria-induced bio-healing concrete leads to a lower filling efficiency than that seen in highly alkaline systems (Khanjani et al., 2021; Li et al., 2013). Its stability field narrows near neutral conditions, increasing the possibility of partial dissolution. Ettringite appears as fine needles with moderate thickness and uniform distribution, suggesting balanced mineral growth under neutral conditions. Such conditions may locally occur in carbonated zones or water-exposed cracks. This leads to weaker ettringite infill and lower bio-healing performance (Suzuki-Muresan et al., 2018). At very high pH levels, the microstructure becomes denser and more compact. Well-formed rhombohedral calcite crystals can be seen (Bots et al., 2011). Under these alkaline conditions, mixed phases of calcite and amorphous calcium carbonate may form when urea hydrolysis is significant, resulting in aggregates or blocky crystals (Zehner et al., 2020; Zehner et al., 2021). Calcite, with its rhombohedral structure, is the most stable and advised phase for long-term crack healing. Ettringite may steadily form in very alkaline environments (Thirupathi et al., 2025). Sulfate and aluminum ions are highly soluble at high pH levels. As a result, ettringite crystals may develop into long, thick, interconnected needles. This enhances early-stage healing and fills gaps. Strong ettringite needles, which physically fill in cracks, and calcite deposits, which provide a dense and stable sealing, improve the concrete’s mechanical strength (Zehner et al., 2021). Therefore, in microbially induced calcium carbonate precipitation (MICP) systems, effective pH control by careful selection of bacterial strains and regulation of urea dosing is required to assure the desired crystal morphology, optimize precipitation rates, and achieve enduring healing (Seidel et al., 2025; Tobler et al., 2016). At low pH, nucleation and crystal growth are strongly influenced by environmental conditions, particularly pH and the supersaturation level of calcium and carbonate ions. These factors control crystal development, polymorph type, and final crystal habit, including size and shape (Oral and Ercan, 2018). Several negative effects occur when the pH drops: it limits the availability required for ettringite growth by decreasing aluminum solubility; concurrently, ettringite becomes unstable and tends to break down into phases like gypsum or aluminate gel (Germishuizen et al., 2018; Trentin et al., 2022). At low pH, ettringite dissolves incongruently, forming gypsum and aluminum hydroxide, reflecting the inhibited availability of aluminum and the destabilization of the phase, limiting its availability for ettringite formation; ettringite becomes unstable and begins to break down, turning into monosulfate or other phases; and crystal growth is inhibited, resulting in the development of shorter, thinner, and incomplete crystals (Tobler et al., 2016). Early growth at low pH frequently results in morphology with irregular, cauliflower-like structures, which reflect the hampered and disordered crystallization process (Oral and Ercan, 2018).

#### 4.1.2 Salinity exposure

Sodium chloride (NaCl) has a major effect on the development of calcium carbonate (CaCO_3_) crystals and the associated MICP process. NaCl alters the bacterial metabolism microenvironment and the local ionic strength, which affects the crystal size and structure of CaCO_3_ (Wang et al., 2014a). At low concentrations, calcite usually forms in its normal rhombohedral habit, and NaCl has only a small inhibitory effect (Achal et al., 2011a; Kasimu and Gu, 2024). Ureolytic or sulfate-reducing bacteria can also encourage the precipitation of ettringite as a secondary healing phase in addition to calcite in bacterial bio-healing concrete, which successfully seals microcracks and restores mechanical performance. However, ettringite production and morphology are significantly impacted by NaCl, particularly in marine environments. The majority of bacterial strains survive at low NaCl exposure, allowing for synergistic crack healing by both calcite and ettringite. The morphology of ettringite crystals is very similar to that of the control system (longer, thin needles with a high aspect ratio) (Hou et al., 2018). At moderate NaCl concentrations, the crystallization process of ettringite and calcium carbonate (CaCO_3_) is considerably changed. Ion competition leads to the formation of smaller and more frequently aggregated calcite crystals, as Cl^−^ adsorption modifies the surface energy of growing crystal faces, while Na^+^ and Ca^2+^ compete for binding site. While slowing crystal formation, increased ionic strength promotes nucleation (Dong et al., 2024; Knight, 2024). Calcium, aluminum, and sulfate ion interactions typically result in ettringite formation in cementitious systems. These reactions result in long, rod-like crystals that improve microstructural integrity by filling up crack voids and expanding. Bacteria can further facilitate this process by altering the local chemical environment, such as by increasing pH or supplying additional ions that promote mineral formation (De Muynck et al., 2010b; Van Belleghem et al., 2016). However, the availability of sulfate and aluminum is disrupted by chloride ions (Cl^−^) from NaCl, resulting in thinner, less crystalline ettringite needles with less expansion and volume, which decreases its ability to heal cracks (De Muynck et al., 2010b; Van Belleghem et al., 2016). This interference leads to partial substitution of ettringite by chloride-bearing AFm phases such as Friedel’s and Kuzel’s salts, often resulting in shorter, thicker ettringite crystals with lower aspect ratios (Balonis and Glasser, 1996). Microbially precipitated CaCO_3_ is more important for healing than ettringite because, absent halotolerant strains, bacterial activity may decline in salt-rich environments (Wang et al., 2024). Ettringite and calcium carbonate (CaCO_3_) exhibit profoundly altered crystallization behavior at elevated NaCl concentrations. High chloride levels cause bacterial cells to experience osmotic stress, which inhibits their development and urease activity. This, in turn, reduces their capacity to raise the local pH and, as a result, inhibits CaCO_3_ precipitation (Fan et al., 2024). Consequently, precipitation frequently moves toward metastable polymorphs like vaterite or aragonite, or even amorphous phases with deformed and irregular morphologies, and stable calcite production is prevented (Dikshit et al., 2022). The chemistry of the pore solution is simultaneously changed by the presence of chloride ions (Cl^−^), which compete with sulfate ions (SO_4_^2-^) to reduce the availability of sulfate, which is necessary for ettringite formation (Dong et al., 2024; Knight, 2024). In addition to interfering with nucleation and elongation, this rivalry encourages the production of substitute chloride-bearing phases, including Friedel’s salt, which further diminishes the degree of ettringite growth. As a result, the strong needle-like shape seen under chloride-free conditions is replaced by shorter, thinner, or incomplete ettringite crystals. These reductions in thickness and volume directly impair the efficiency of bacterial bio-healing concrete by reducing crack-sealing capacity and compromising long-term durability in saline environments (Khanjani et al., 2021; Wang et al., 2024).

### 4.2 Compressive strength

#### 4.2.1 pH

The improvement in compressive strength of specimens containing bacteria immobilized on nano-eggshells compared to control specimens is attributed to accelerated hardening due to increased surface area and strong electrostatic interactions. The observed enhancement in compressive strength can be attributed to the formation of calcium carbonate. Specifically, the calcium carbonate content increased by 12.5% and 16.4% under curing conditions at pH 9 and pH 11, respectively. Strength was increased by combining eggshell nanoparticles with bacteria, and the combined effect of both led to the closure of cracks. The eggshell nanoparticles then functioned as a filler product and helped create a tighter microstructure because of their ultrafine size. Furthermore, the immobilizing medium with nanoscale size was effective for the uniform dispersion of microorganisms in the matrix, effectively sealing nano/micro cracks (Mahmood et al., 2022; Seifan et al., 2018; Shaheen et al., 2019). EShN increased the durability of concrete since it contains a lot of calcium oxide, which is necessary for hydration in concrete production. Additionally, it reduces water absorption as a result; this concrete might be very resistant to environmental stresses such as those caused by acids and sulfates (Murthi et al., 2022). EShN increases the compressive strength of concrete throughout all age ranges. During hydration, CaCO_3_ joins forces to form an enhanced hydration process (Lavanya et al., 2023). Hussein et al. (2020) demonstrated that nano-eggshell powder was utilized to increase the cement mortar’s compressive strength. At a later age, it was observed that CaCO_3_ improves the mechanical properties of cement mortar. It has been demonstrated that adding bacteria to concrete, especially when it is cured in an alkaline environment, increases compressive strength by causing microbially induced calcium carbonate precipitation (MICP). In order to fill in microcracks and create a denser, more durable structure, bacteria in the concrete matrix precipitate calcium carbonate (CaCO_3_) (Cheng et al., 2013; Hou et al., 2018; Wang et al., 2014b). Alkaline environments are ideal for cement hydration. A stronger microstructure results from maintaining the stability of calcium silicate hydrate (C-S-H) gel and the solubility of calcium hydroxide (CH) at high levels of pH (Nasvik and Smith, 2022). At high pH (>9), bicarbonate ions (HCO_3_^−^) are converted into carbonate ions (CO_3_^2-^). Ettringite crystals can develop into long, thick needles that form interconnected networks at high pH values because sulfate and aluminum ions are extremely soluble at these levels. This helps to fill gaps and promote early-stage healing; this increases the compressive strength (Zehner et al., 2021). Compressive strength decrease in lower pH environments because of leaching of calcium hydroxide can occur, weakening the concrete structure. A decrease in pH leads to several detrimental effects, including reduced aluminum solubility, which limits the availability required for ettringite growth. Simultaneously, ettringite becomes unstable and tends to decompose into phases such as gypsum or aluminate gel (Germishuizen et al., 2018; Trentin et al., 2022). At low pH, ettringite dissolves incongruently, forming gypsum and aluminum hydroxide, reflecting the inhibited availability of aluminum and the destabilization of the phase, limiting its availability for ettringite formation (Wang et al., 2014a). On the other hand, settings with an acidic or neutral pH can interfere with these hydration products, reducing their strength (Oral and Ercan, 2018). Concrete’s pH changes over time as a result of carbonation, a series of chemical processes that causes the pH to drop. A carbonated layer of concrete results from the CO_2_ mostly penetrating from the surface through a diffusion mechanism. Since the majority of alkaliphilic bacteria do not grow at pH values lower than 9, bio-healing should be sluggish or possibly nonexistent if the concrete carbonates quickly (Horikoshi, 2011). The rate at which the microbiologically produced precipitation accelerates varies depending on the conditions. After 28 days of curing, the compressive strength of the samples decreased by only 3%. It is evident that mortar cubes' characteristics and healing process change when the pH level changes. All the preliminary studies reveal the compatibility of bacterial species that survive in an alkaline environment with a mortar-rich environment, which adds alkalinity to the cement mortar bacterial mix as a whole. So when they are exposed to acidic and other neutral conditions, the alkalinity still exists, and a pH ratio is stabilized by accelerating the growth of bacterial species (Subash et al., 2019). According to Jena et al. (2020b), an active bacterial cell referred to as a vegetation cell can transform into a spore when subjected to high pH (12–13).

#### 4.2.2 Salinity

The specimens with immobilized bacterial cells cured at salinity concentrations of had a higher compressive strength than their corresponding control specimens without bacteria subjected to the same salinity concentrations. Microbially induced calcium carbonate precipitation (MICP) is a natural process that uses bacteria to help create calcium carbonate (CaCO_3_) in concrete. This process has been used to improve the mechanical properties of concrete by forming calcium carbonate bonds with the concrete matrix. The procedure can help to improve the overall bonding strength between the aggregate and the cementitious phase, resulting in an increase in compressive strength (Jhatial et al., 2019; Tziviloglou et al., 2015). MICP, which fills micro-cracks and improves mechanical characteristics, is responsible for an increase in compressive strength. The combination comprising EShN has a high early compressive strength due to the highly specific surface areas (Khushnood et al., 2019). Salt negatively impacts both bacterial survival and cement hydration processes, which is the main cause of the reduction in compressive strength of bacterial concrete cured in saline conditions. Calcium carbonate, which fills cracks and increases strength, is frequently precipitated by bacteria in bio-healing concrete. High salinity levels, however, can produce a hypertonic environment that dehydrates and inactivates these microorganisms. The desired bio-healing effect is weakened by this decrease in microbially induced calcite precipitation and this decreased compressive strength (Achal et al., 2011b). Chloride ions derived from salt can alter the cement hydration process by inhibiting the formation of calcium silicate hydrate (C–S–H), the primary strength-giving phase, or by forming non-strength-contributing compounds. As a result, the matrix becomes less dense, reducing the compressive strength (Achal et al., 2011b; Debnath and Debnath, 2020). Salt can cause osmotic pressure gradients within the concrete, leading to microcracks or expansion over time, especially when salts are absorbed and crystallize within pores. In bacteria-containing concrete, the lack of effective healing due to inactive bacteria means these microcracks persist and grow, reducing strength further. Salts can prevent calcium carbonate or other biominerals from precipitating by altering pH, adding competing ions (such as sodium or magnesium), or creating soluble complexes that prevent precipitation. This interference with the calcium carbonate precipitation caused by microbes jeopardizes the concrete bio-healing. The compressive strength also decreases because ettringite crystals become shorter, thinner, or incomplete. These reductions in thickness and volume directly impair the efficiency of bacterial bio-healing concrete, as insufficient ettringite formation diminishes crack-sealing capacity and compromises long-term durability in saline environments (Balonis and Glasser, 1996; Wang et al., 2024). The chemistry of the pore solution is simultaneously changed by the presence of chloride ions (Cl^−^), which compete with sulfate ions (SO_4_^2-^) to reduce the availability of sulfate, which is necessary for ettringite formation (Dong et al., 2024; Knight, 2024). A study by Yang and Wang examined the effectiveness of microbially induced calcium carbonate precipitation (MICP) under high salinity and low oxygen conditions using microfluidic techniques. Their studies revealed that high salinity concentration reduces bacterial attachment and calcium carbonate crystal formation, thereby impacting the MICP process. Specifically, in seawater environments, high salinity and cold temperatures significantly affected calcium carbonate crystal shape and type, with high salinity reducing crystal quantity by 20.2%. This reduction in crystal formation is indicative of decreased bacterial metabolic activity under saline conditions (Yang and Wang, 2024).

### 4.3 Flexural and tensile strength

#### 4.3.1 pH

The flexural and tensile strength increased for the concrete containing immobilized bacterial cells when compared to their relative controls, which were without bacteria and subjected to the same conditions. The samples' continuous rise in tensile strength and flexural strength suggests that microbial activity might improve the concrete matrix’s internal cohesiveness. It is known that biomineralization, especially the precipitation of calcium carbonate, fills holes and microcracks, which may explain the noted increases in tensile strength (Ibrahim et al., 2024). Concrete’s developing tensile strength could be explained by the creation of a dense interfacial transition zone (ITZ) between the natural aggregate and cement. The nanoparticles improve the matrix-aggregate bond because of their large specific surface area (Amin et al., 2022). Eggshell nanoparticles used as a bacterial carrier represent a promising and sustainable material, as they are waste-derived and enhance the mechanical properties of composites, improving strength and stiffness while providing a low-cost immobilization medium. The increased flexural strength and tensile strength of bacterial concrete cured in alkaline conditions (pH > 7) is primarily due to the ideal conditions for bacterial survival and activity. Many bacteria employed in bio-healing concrete are alkaliphilic, which means they thrive in high pH conditions like concrete pore solutions. Bacteria thrive in alkaline settings and use metabolic mechanisms to precipitate calcium carbonate (CaCO_3_). This precipitation fills pores and cures microcracks, resulting in enhanced flexural strength and tensile strength, particularly during extended curing durations (Khushnood et al., 2019). Bacterial activity is inhibited in acidic settings, resulting in a loss in flexural strength and tensile strength for bacterial concrete cured in pH < 7. Most bacteria employed in bio-healing concrete are alkaliphilic, meaning they cannot survive or function well in acidic or neutral settings. When the pH falls below 7, the metabolic activity responsible for calcium carbonate (CaCO_3_) precipitation is significantly decreased or interrupted. This results in poorer crack healing and poor pore filling. Consequently, the concrete matrix remains more porous and weaker, resulting in reduced flexural strength (Wang et al., 2012).

#### 4.3.2 Salinity

The flexural strength results demonstrated that EShN enhanced the tensile and flexural strength of concrete due to their surface characteristics, which improve cohesion between the cement matrix and filler surface (Lavanya et al., 2023). The finer particle composition of EShN may be the cause of the higher flexural strength (Alemu et al., 2022; Grzeszczyk et al., 2022; Hussein et al., 2014; Jagadesh et al., 2018). High salinity levels can adversely affect the viability and metabolic functions of bacteria, which are crucial for the process of MICP. This process is essential for crack healing and strength enhancement in bacterial concrete (Zhang et al., 2021). Exposure to sodium chloride (NaCl) solutions can cause the development of compounds such as Friedel’s salt and calcium oxychloride within the concrete matrix. These reactions can compromise the integrity of the concrete. Magnesium ions can replace the calcium in portlandite, lower the alkalinity of the pore solution, and eventually destabilize the C-S-H gel. This leads to a loss in flexural strength and tensile strength. The constant formation of salt crystals within the limited regions of concrete pores causes internal stress. When the crystallization pressure surpasses the tensile strength of the concrete, it results in cracking, increased porosity, and degradation of the interfacial transition zones, all contributing to a decline in flexural strength (Yi et al., 2020). Saline conditions introduce ions such as chloride and sulfate, which can interfere with cement’s regular hydration reactions. This interference can cause the production of expansive compounds, increased porosity, and microcracking, all of which reduce the tensile strength of concrete (Qu et al., 2021). The researchers discovered that salt crystallization affects the pore structure of concrete. Initially, porosity reduces, which improves mechanical performance. However, with time, the collection and growth of salt crystals within the pores generate expansive stresses that result in microcracking and increasing porosity. This degradation compromises the integrity of the concrete, leading to a reduction in its flexural strength (Pietruszczak et al., 2022). Findings: After 90 days, concrete cured in high-salinity water had a split tensile strength that was 24.92% lower than that of potable water. Chloride and sulfate ions interfered with hydration and increased porosity, causing the decrease (Kponmwosa et al., 2020). Environmental factors, such as salinity, can affect microbial CaCO_3_ precipitation, reducing bacterial function and concrete strength (Dhami et al., 2013).

## 5 Conclusion

This study shows that the performance of bio-concrete using *Bacillus paramycoides* G10 immobilized on eggshell nanoparticles is strongly influenced by pH and salinity. High pH levels enhance bacterial activity, promote stable calcite and ettringite formation, and significantly improve concrete strength. On the other hand, high salinity leads to less stable mineral phases, bacterial mineralization still provides measurable strength improvement. SEM results confirm denser, more compact microstructures under favorable conditions. Overall, controlling pH and salinity is essential for optimizing MICP and ensuring durable bio-concrete for harsh environments such as marine and coastal structures.

However, several limitations should be acknowledged. The bacterial concrete system shows reduced practicality under dry conditions, where limited moisture availability may restrict bacterial activity. In addition, the self-healing mechanism is mainly effective for small cracks (typically < 0.5 mm) and requires relatively long healing periods ranging from weeks to months, which may limit its applicability in time-sensitive or structural repair applications. Moreover, achieving uniform nutrient distribution remains challenging, and variations in bacterial activity can affect the consistency and reliability of the healing process.

Future research should focus on improving the performance and practical applicability of bacterial concrete systems by enhancing bacterial survival under dry and harsh environmental conditions, accelerating healing kinetics, and expanding the effective crack width range. In addition, further investigations are recommended to evaluate long-term durability under real environmental exposure conditions and large-scale field applications. Future studies should also explore different bacterial strains, optimize nutrient delivery systems to ensure uniform bacterial activity, and assess the influence of environmental factors such as temperature variations and wet–dry cycles to improve the overall reliability.

## Data Availability

The original contributions presented in the study are included in the article/supplementary material, further inquiries can be directed to the corresponding author.
